# Pearson's Dentist's Appointment Book

**Published:** 1888-02

**Authors:** 


PEARSON'S DENTISTS' APPOINTMENT BOOK. By R. J. Pearson, Kansas City, Mo.

This is a very convenient appointment book, of a size to be carried in the vest pocket, and arranged for appointments covering eight hours of every operating day in the year. It is very nicely bound in red Russian leather.

